# Trajectories of energy intake distribution and subsequent risk of hyperglycemia among Chinese adults: findings from the China Health and Nutrition Survey (1997–2018)

**DOI:** 10.1007/s00394-021-02745-3

**Published:** 2021-11-27

**Authors:** Xiaoyun Song, Huijun Wang, Chang Su, Zhihong Wang, Wenwen Du, Haojie Hu, Feifei Huang, Jiguo Zhang, Xiaofang Jia, Hongru Jiang, Yifei Ouyang, Li Li, Jing Bai, Xiaofan Zhang, Gangqiang Ding, Bing Zhang

**Affiliations:** Chinese Center for Disease Control and Prevention, National Institute for Nutrition and Health, Beijing, 100050 China

**Keywords:** Diabetes, Energy intake, Hyperglycemia, Impaired fasting glucose, Multi-trajectory model

## Abstract

**Aims:**

Few studies have examined the secular trend of the energy intake distribution, and its effect on future risk of hyperglycemia. This study aims to describe trajectories of energy intake distribution over 12 years and relate them to subsequent risk of hyperglycemia over 9 years of follow-up.

**Methods:**

Our study used ten waves of data from the CHNS survey, a population-based longitudinal survey in China, ongoing since 1989. We examined a cohort of adult participants who were free from diabetes but had at least three waves of dietary data from 1997 to 2009. We assessed energy intake using three consecutive 24 h recalls. We used these data to identify trajectory groups of energy intake distribution by multi-trajectory model based on energy intake proportions of breakfast, lunch, and dinner. We followed up participants for hyperglycemia, diabetes, and impaired fasting glucose for 9 years from 2009 to 2018. Outcomes were ascertained with fasting glucose, serum HbA1c, and self-report of diabetes and/or glucose-lowering medication. We estimated relative risk (RR) for hyperglycemia, diabetes, and impaired fasting glucose by identified trajectory groups using multilevel mixed-effects modified Poisson regression with robust (sandwich) estimation of variance. Gender difference was additionally examined.

**Results:**

A total of 4417 participants were included. Four trajectory groups were identified, characterized and labeled by “Energy evenly distributed with steady trend group” (Group 1), “Dinner and lunch energy dominant with relatively steady trend group” (Group 2), “Dinner energy dominant with increasing trend and breakfast energy with declining trend group” (Group 3), and “breakfast and dinner energy dominant with increasing trend group” (Group 4). During 48,091 person-years, 1053 cases of incident hyperglycemia occurred, 537 cases of incident diabetes occurred, and 516 cases of impaired fasting glucose occurred. Compared with Group 1, Group 3 was associated with higher subsequent risk of incident hyperglycemia in 9 years of follow-up (RR = 1.28, 95% CI = 1.02, 1.61). No association was found for incident diabetes and impaired fasting glucose. Among males, Group 3 was associated with higher risk of incident hyperglycemia in 9 years of follow-up (RR = 1.44, 95% CI = 1.07, 1.94). No relationship was found in females.

**Conclusions:**

Energy intake distribution characterized by over 40% of energy intake from dinner with a rising trend over years was associated with higher long-term risk of hyperglycemia in Chinese adults.

**Supplementary Information:**

The online version contains supplementary material available at 10.1007/s00394-021-02745-3.

## Introduction

Diabetes is a risk factor for cardiovascular disease, mortality and many other adverse health consequences [1]. Hyperglycemia is one of the diagnostic features of diabetes or pre-diabetes, which includes impaired fasting glucose (IFG) and impaired glucose tolerance (IGT). Strong evidence confirm that hyperglycemia increases the risk of mortality, cardiovascular disease, chronic kidney disease, retinopathy, and neuropathy [2–6], compared with normoglycemia.

The high prevalence of diabetes can be prevented by changes in lifestyle of which dietary factors play a vital role [7]. In addition to the effects of specific nutrients, foods, and dietary patterns, accumulating evidence suggest that meal timing is a key factor in the control of glucose metabolism [8]. Prospective studies [9, 10] examining the association between meal timing or meal pattern and diabetes have mainly focused on isolated meal, e.g., breakfast or dinner, which do not capture the full spectrum of meal pattern across the day. Some previous studies [11–13] have assessed eating pattern based on energy contribution of meals in the day. Using latent class analysis, three distinct temporal eating patterns of Australia adults were identified based on whether or not an eating occasion occurred within each hour of the day [11], while four distinct temporal eating patterns of US adults were identified based on energy contribution using kernel K-Means Clustering method [12, 13]. However, these cross-sectional studies could not examine the secular trend of eating pattern, and the relationship between eating pattern based on energy intake distribution and risk of diabetes remains unclear. Since change of meal patterns over time has not been elucidated by previous research [14] and whether meal pattern has a long-term effect on health is rarely examined, studying the secular trend of energy intake distribution over time in relation to subsequent risk of hyperglycemia might reveal more informative associations.

Therefore, we used repeated data on energy intake of meals to examine different trajectories of daily energy intake distribution, and their subsequent risks of incident hyperglycemia in a population-based cohort setting.

## Research design and methods

### Study design

Our study used data from the China Health and Nutrition Survey (CHNS), a population-based longitudinal survey in China. The original survey in 1989 used a multistage, random cluster design in eight provinces to select a stratified probability sample. Specifically, two cities and four counties per province were selected. Within cities, two urban and two suburban communities were randomly selected. Within counties, one community in the capital city and three rural villages were randomly chosen. Twenty households per community were randomly selected within each community. All family members in the household were invited to participant. Since 1993, all new households formed by individuals within the sample households were added to the sample. In addition, from 1997 onward new households and communities were added to replace those lost in the previous wave of survey. In each follow-up survey, individual dietary and anthropometric data, as well as household- and community-level socio-economic data were obtained. Blood samples were collected in 2009, 2015, and 2018 surveys. More details regarding the CHNS are provided in the previous article [15].

Our study included participants aged over 18 years who did not have diabetes but had data for dietary intake at meals at more than 3 rounds during 1997–2009. We used these data to identify trajectories of daily energy intake distribution based on proportions of energy intake from three main meals. We followed up participants for incident hyperglycemia, including diabetes and IFG, from 2009 to 2018. Data for incidence of self-reported diabetes were collected from round 1997 onward in the CHNS, but we performed subsequent analyses from 2009 round onward to (1) reduce a possible trajectory classification bias caused by concomitant onset of diabetes during the period when energy intake distribution trajectories were assessed, and (2) investigate association between energy intake distribution trajectory groups and pre-diabetes, since fasting glucose was only collected from round 2009 onward.

### Study population

In the longitudinal cohort, participants aged ≥ 18 years, not during pregnant or lactating period, with at least 3 waves of dietary data between 1997 and 2009 and visited at least 2 waves between 2009 and 2018 with complete fasting glucose measurements were eligible for inclusion (*n* = 5697). Participants who ever self-reported diabetes from 1997 to 2009 (*n* = 252), and who were diagnosed with diabetes and/or IFG when they first entered the cohort from 2009 to 2018 (*n* = 734) were excluded. The final analytical sample included 4711 participants. The main reason for drop-out of participants was moving out of the original community with the process of urbanization. Figure S1 in supplemental materials presents the flow chart of participant selection. Table S1 in supplemental materials compares characteristics between included and excluded participants.

### Calculation of proportions of energy intake from breakfast, lunch and dinner

In the CHNS, dietary data were collected based on a combination of three consecutive 24-h recalls (two weekdays and 1 weekend) at the individual level and a food inventory at the household level during the same three-day period. Information on types and amounts of food consumed at each meal during the previous 24 h were recorded by well-trained field interviewers. In our study, amounts of every condiment (such as oil, salt, soy sauce) collected by the weighing and measuring technique at the household level was firstly allocated to each individual, then to each recall day, and finally to each meal, according to the proportion of energy assessed by 3–24-h recall at the individual, day, and meal level, respectively. Energy intake from both food and condiment at each meal was calculated by the China Food Composition. In the present study, breakfast, lunch, and dinner were classified as main meals. The proportions of energy intake at breakfast, lunch and dinner were firstly calculated for each recall day, then proportions of energy intake at breakfast, lunch and dinner were averaged across their consumption days to obtain mean estimates, respectively.

### Outcome measures

Overnight fasting blood samples were collected by trained nurses and biochemical indexes were measured in a national lab in Beijing with strict quality control. Fasting plasma glucose concentration was measured by the GOD-PAP (Randox Laboratories Ltd., London, UK) method. HbA1c were measured by a high-performance liquid chromatography system (model HLC-723 G7; Tosoh Corporation, Tokyo, Japan).

Diabetes was defined as self-report of diabetes (diagnosed by doctors and/or taking glucose-lowering medication including oral medicine and injection of insulin) and/or fasting blood glucose ≥ 7.0 mmol/L and/or HbA1c ≥ 48 mmol/mol (6.5%) [16]. IFG was defined as fasting blood glucose level of 6.1–6.9 mmol/L [16]. Hyperglycemia was defined as diabetes and/or IFG in our study. Data for incidence of self-reported diabetes and glucose-lowering medication were collected from 1997 round onward. Serum glucose and whole HbA1c were measured in round 2009, 2015, and 2018.

### Assessment of covariates

We assessed covariates between 2009 and 2018. The following measures were considered covariates: age; gender; educational level [low (i.e., completed primary school), medium (i.e., completed middle school), high (i.e., completed high school and above)]; geographic region (urban and rural); total physical activity [high (i.e., total physical activity days ≥ 7 days/week and total physical activity level ≥ 3000 METs-min/week), medium (i.e., total physical activity days ≥ 5 days/week and total physical activity level ≥ 600 METs-min/week), and low (i.e., physical activity not meeting the above medium and high grouping criteria) [17]]; sleep duration (< 6 h, 6–9 h, and > 9 h); smoking (non-smoker and current smoker); alcohol drinking (non-drinker and current drinker); annual per capita household income; community urbanicity index, calculated based on 12 multi-dimensional components including physical, social, cultural and economic environment of the community [18]; total energy intake; CDGI (2019)-A score, calculated based on 13 food-related components and 1 nutrient-related component reflecting compliance for meeting the Chinese Dietary Guidelines 2016 [19]; body mass index (BMI); waist circumference (WC); systolic blood pressure (SBP); and diastolic blood pressure (DBP).

### Statistical analysis

We used a group-based multi-trajectory model [20] to identify trajectory groups of daily energy intake distribution based on proportions of energy intake from breakfast, lunch, and dinner. Group-based multi-trajectory model is an extension of the univariate group-based trajectory modeling (GBTM), which defines trajectory groups in terms of trajectories for multiple indicators (herein energy intakes from breakfast, lunch, and dinner) not just one indicator [20]. As a result, each identified trajectory group captured distinct patterns of energy intake distribution with temporal co-dependencies of three meals, which allowed us to represent the longitudinal course of energy intake of three main meals jointly. Models with 1–5 trajectory groups using censored normal distribution with a quartic trajectory function were fit firstly to determine the optimal group number. We did not go beyond 5 groups for the sake of parsimony. The optimal group number was determined using model-adequacy criteria [21] including the logged Bayes factor (≈2ΔBIC, > 10), average posterior probability of assignment (APPA, > 0.70), odds of correct classification (OCC, > 5 for all groups), and proportion of individuals estimated to be assigned to each group (≥ 1% for each group). After settling the number of group number, the polynomial orders of each trajectory were selected, based on statistical significance. Each participant was finally classified into a group where the posterior probability of trajectory group membership calculated by multi-trajectory model was the largest.

Baseline demographic, lifestyle, and anthropometric variables were compared among the identified trajectory groups. Analysis of variance was used for continuous variables with normal distribution. Kruskal–Wallis test was used for continuous variables with non-normal distribution and Chi-square test were used for categorical variables.

To evaluate the association between energy intake distribution trajectory group of and subsequent incident hyperglycemia in 9 years of follow-up, we chose modified Poisson regression with robust (sandwich) estimation of variance, which is a reliable approach to estimate relative risk directly with binary outcomes [22], rather than logistic regression. Due to the prospective design of our study, we considered the relative risk was preferred over the odds ratio. Besides, odds ratio yielded from logistic regression is not appropriate to estimate relative risk when the rare disease assumption is violated. Specifically, a three-level mixed-effects modified Poisson regression with robust (sandwich) estimation of variance was used. In this analysis, we took household as the third level, individual as the second level, and repeated measurement of individual as the first level, to count for the clustering sampling method used in CHNS, and the repeated measures within individuals. We also performed additional analysis stratified by types of outcomes and by gender. For all analysis, 4 models were fitted: Model 1 adjusted for no covariates. Model 2 adjusted for age, gender, marriage status, an education level, geographic region, annual per capita household income, urbanicity index, physical activity, smoking, alcohol drinking, sleep duration, and chronic disease history. Model 3 additionally adjusted for total energy intake and CDGI (2019)-A score. Model 4 additionally adjusted for BMI, WC, SBP, and DBP.

To assess the robustness of trajectory classification and the associated risk of hyperglycemia across groups, we did sensitivity analyses in participants with 3–5 rounds of dietary data on meals from 1997 to 2009 and visited all 4 rounds from 2009 to 2018. We reassessed trajectory groups and examined their associations with subsequent risk of hyperglycemia in 9 years of follow-up.

All the analyses were conducted in SAS 9.4 (SAS Institute, Inc., Cary, NC, USA) and Stata 15SE (StataCorp., College Station, TX, USA). Group-based multi-trajectory model was conducted by package TRAJ for Stata [20]. *P* < 0.05 was considered statistically significant.

## Results

### Identifying latent trajectory groups of energy intake distribution

**Fig. 1:**
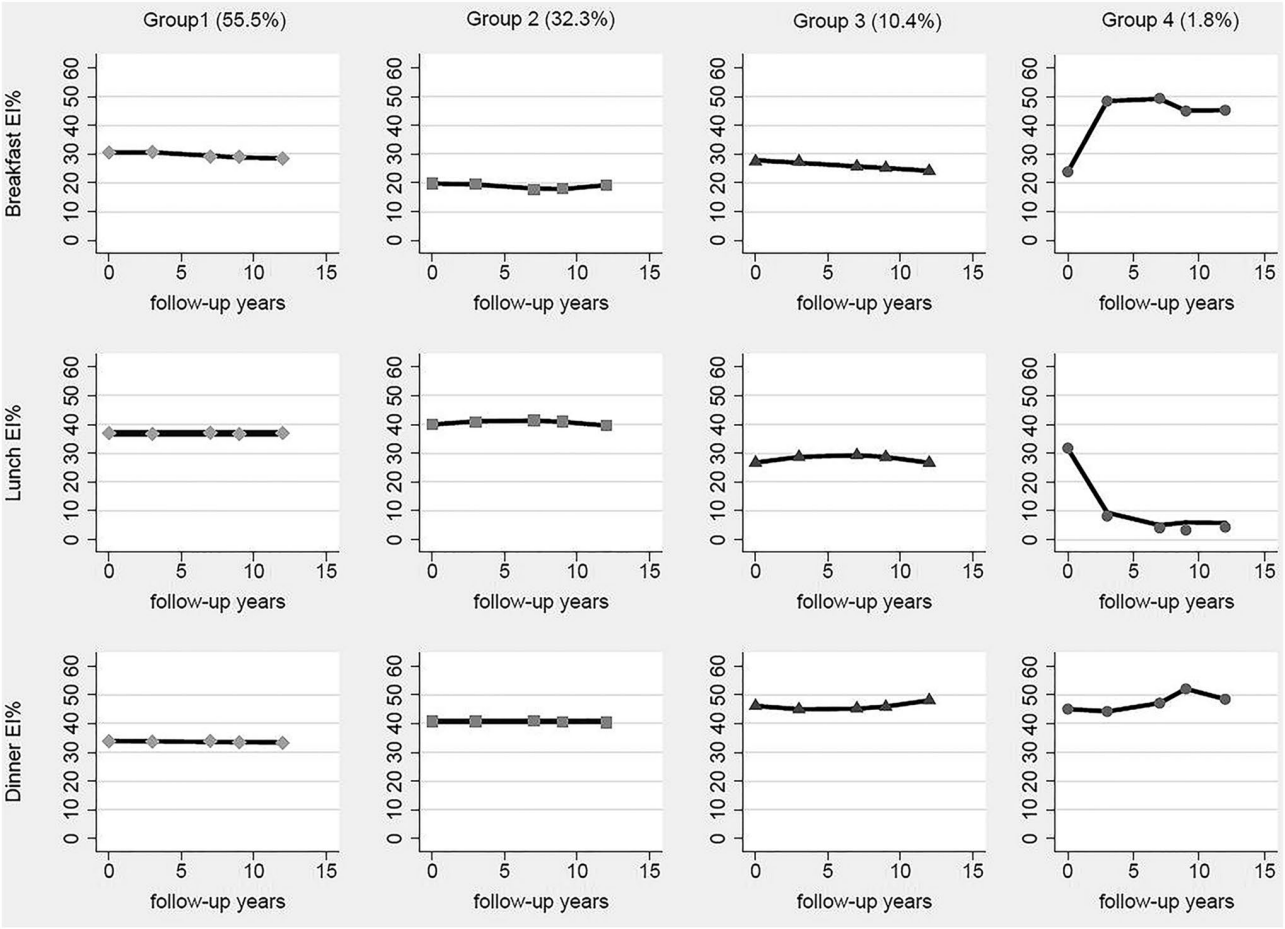
Estimated trajectory groups of energy intake distribution among Chinese adults. Lines with a diamond represent Group 1, labeled “Energy evenly distributed with steady trend group”; Lines with a square represent Group 2, labeled “Dinner and lunch energy dominant with relatively steady trend group”; Lines with a triangle represent Group 3, labeled “Dinner energy dominant with increasing trend and breakfast energy with declining trend group”; Lines with a point represent Group 4, labeled “breakfast and dinner energy dominant with increasing trend group”

According to model-adequacy criteria, the goal of parsimony, and the rule of interpretability, we chose the 4-group solution for all participants (Fig. 1). Table S2 in supplemental materials presents parameters of model-adequacy criteria.

The first trajectory group comprised 55.5% of the participants, characterized by steady trends of EI% from three main meals, which were about 30% from breakfast, 40% from lunch, and 30% from dinner. Thus, this group was labeled “Energy evenly distributed with steady trend group”. The second trajectory group comprised 32.3% of the participants, characterized by a lower EI% of about 20% from breakfast, and higher EI% of about 40% from lunch and 40% EI from dinner. Besides, a slightly downward trend of lunch EI% and a slightly fluctuation of breakfast EI% were observed. Taken together, this group was labeled “Lunch and dinner energy dominant with relatively steady trend group”. The third trajectory group comprised 10.4% of the participants, characterized by a relatively higher EI% from dinner which was 40–50% with an increasing trend. At the same time, EI% from breakfast was about 20–30% with a clear declining trend, and EI% from lunch was about 30% with a slightly downward trend. Therefore, this group was labeled “Dinner energy dominant with increasing trend and breakfast energy with declining trend group”. The fourth trajectory group comprised 1.8% of the participants, characterized by a sharp declining trend of EI% from lunch, and a clearly upward trend of EI% from breakfast and a high EI% from dinner. Therefore, this group was labeled “breakfast and dinner energy dominant with increasing trend group”. The trajectory groups of energy intake distribution among males and females are presented in Figure S2 in supplemental materials, which were similar to that of all participants.

### Baseline characteristics of the cohort by the four estimated latent trajectory groups

In the baseline between 2009 and 2018, participants in Group 1 were often females, less likely a current smoker, with medium-to-high level of physical activity, and had higher baseline mean level of WC and DBP. Participants in Group 2 had higher education level, longer sleep duration, and mean level of urbanicity score, but lower proportion of medium-to-high level of physical activity. Participants in Group 3 had higher proportions of males and current drinker, were less likely married and with lower baseline mean level of BMI, WC, and SBP. Participants in Group 4 had higher proportions of current smoker, with lower education level, shorter sleep duration, lowest mean level of urbanicity score and highest baseline mean level of BMI and SBP (Table 1).

**Table 1:** Baseline characteristics by the four estimated latent trajectory groups

Baseline characteristics	Group 1	Group 2	Group 3	Group 4	*P* value
	(*N* = 2614)	(*N* = 1523)	(*N* = 490)	(*N* = 84)	
Age (year, mean [SD])	53.40 (12.31)	54.21 (12.35)	53.30 (12.79)	56.15 (12.48)	0.05
Gender (%)					
Man	1147 (43.88)	758 (49.77)	244 (49.80)	39 (46.43)	0.001
Females	1467 (56.12)	765 (50.23)	246 (50.20)	45 (53.57)	
Marriage status (%)					
In marriage	2341 (89.56)	1345 (88.31)	416 (84.90)	72 (85.71)	0.02
Other status	273 (10.44)	178 (11.69)	74 (15.10)	12 (14.29)	
Education level (%)					
Primary school	1250 (47.82)	688 (45.17)	257 (52.45)	61 (72.62)	< 0.001
Middle school	867 (33.17)	497 (32.63)	177 (36.12)	21 (25.00)	
High school and above	497 (19.01)	338 (22.19)	56 (11.43)	2 (2.38)	
Geographic region (%)					
Urban	589 (22.53)	556 (36.51)	153 (31.22)	6 (7.14)	< 0.001
Rural	2025 (77.47)	967 (63.49)	337 (68.78)	78 (92.86)	
Physical activity (%)					
Low	2274 (86.99)	1419 (93.17)	437 (89.18)	74 (88.10)	< 0.001
Medium	222 (8.49)	75 (4.92)	37 (7.55)	6 (7.14)	
High	118 (4.51)	29 (1.90)	16 (3.27)	4 (4.76)	
Sleep duration (%)					
6–9 h	2277 (87.11)	1316 (86.41)	436 (88.98)	70 (83.33)	< 0.001
< 6 h	77 (2.95)	24 (1.58)	10 (2.04)	7 (8.33)	
> 9 h	260 (9.95)	183 (12.02)	44 (8.98)	7 (8.33)	
Smoking (%)					
Nonsmoker	1931 (73.87)	1057 (69.40)	338 (68.98)	56 (66.67)	0.005
Current smoker	683 (26.13)	466 (30.60)	152 (31.02)	28 (33.33)	
Alcohol drinking (%)					
Nondrinker	1801 (68.90)	985 (64.67)	307 (62.65)	64 (76.19)	0.002
Current drinker	813 (31.10)	538 (35.33)	183 (37.35)	20 (23.81)	
Chronic disease history (hypertension or myocardial infarction or apoplexy or cancer, %)					
No	2270 (86.84)	1334 (87.59)	439 (89.59)	72 (85.71)	0.373
Yes	344 (13.16)	189 (12.41)	51 (10.41)	12 (14.29)	
Per capita household income (yuan/year, median [IQR])	2,2468 (11,583,40,412)	2,4382 (11,586,45,214)	2,0612 (11,437,33,421)	2,4611 (12,991,36,963)	0.001
Urbanicity score (mean [SD])	62.35 (18.51)	70.88 (18.68)	58.22 (16.80)	56.55 (9.86)	< 0.001
BMI (mg/kg, mean [SD])	23.62 (3.34)	23.12 (3.55)	22.18 (3.25)	25.19 (18.63)	< 0.001
WC (cm, mean [SD])	83.93 (9.92)	81.79 (9.74)	78.83 (9.85)	78.87 (9.99)	< 0.001
SBP (mmHg, mean [SD])	125.93 (18.78)	124.09 (18.31)	124.04 (17.74)	135.54 (22.39)	< 0.001
DBP (mmHg, mean [SD])	81.73 (10.94)	79.71 (11.44)	79.18 (10.91)	78.99 (13.24)	< 0.001
CDGI (2019)-A score (mean [SD])	45.47 (11.61)	45.24 (10.78)	45.82(10.10)	47.14 (10.93)	0.3912
Total energy intake (kcal, mean [SD])	2351.47 (749.75)	2338.44 (737.23)	2253.92 (691.20)	2379.41 (777.99)	0.059
Breakfast EI% (mean [SD])	28.58 (7.93)	19.65 (9.75)	24.31 (9.21)	44.28 (9.53)	< 0.001
Lunch EI% (mean [SD])	36.85 (8.65)	38.93 (8.39)	26.64 (10.27)	6.76 (14.36)	< 0.001
Dinner EI% (mean [SD])	33.31 (6.99)	40.04 (7.81)	47.95 (9.50)	47.53 (8.59)	< 0.001

Total physical activity [high (i.e., total physical activity days ≥ 7 days/week and total physical activity level ≥ 3000 METs-min/week), medium (i.e., total physical activity days ≥ 5 days/week and total physical activity level ≥ 600 METs-min/week), and low (i.e., physical activity not meeting the above medium and high grouping criteria) [17]]; community urbanicity index, calculated based on 12 multi-dimensional components including physical, social, cultural and economic environment of the community [18]; BMI, body mass index; SBP, systolic blood pressure; DBP, diastolic blood pressure; CDGI (2019)-A score, calculated based on 13 food-related components and 1 nutrient-related component reflecting compliance for meeting the Chinese Dietary Guidelines 2016 [19]; EI%, percentage of total energy intake

### Longitudinal association between trajectory groups of energy intake distribution and hyperglycemia

**Fig. 2:**
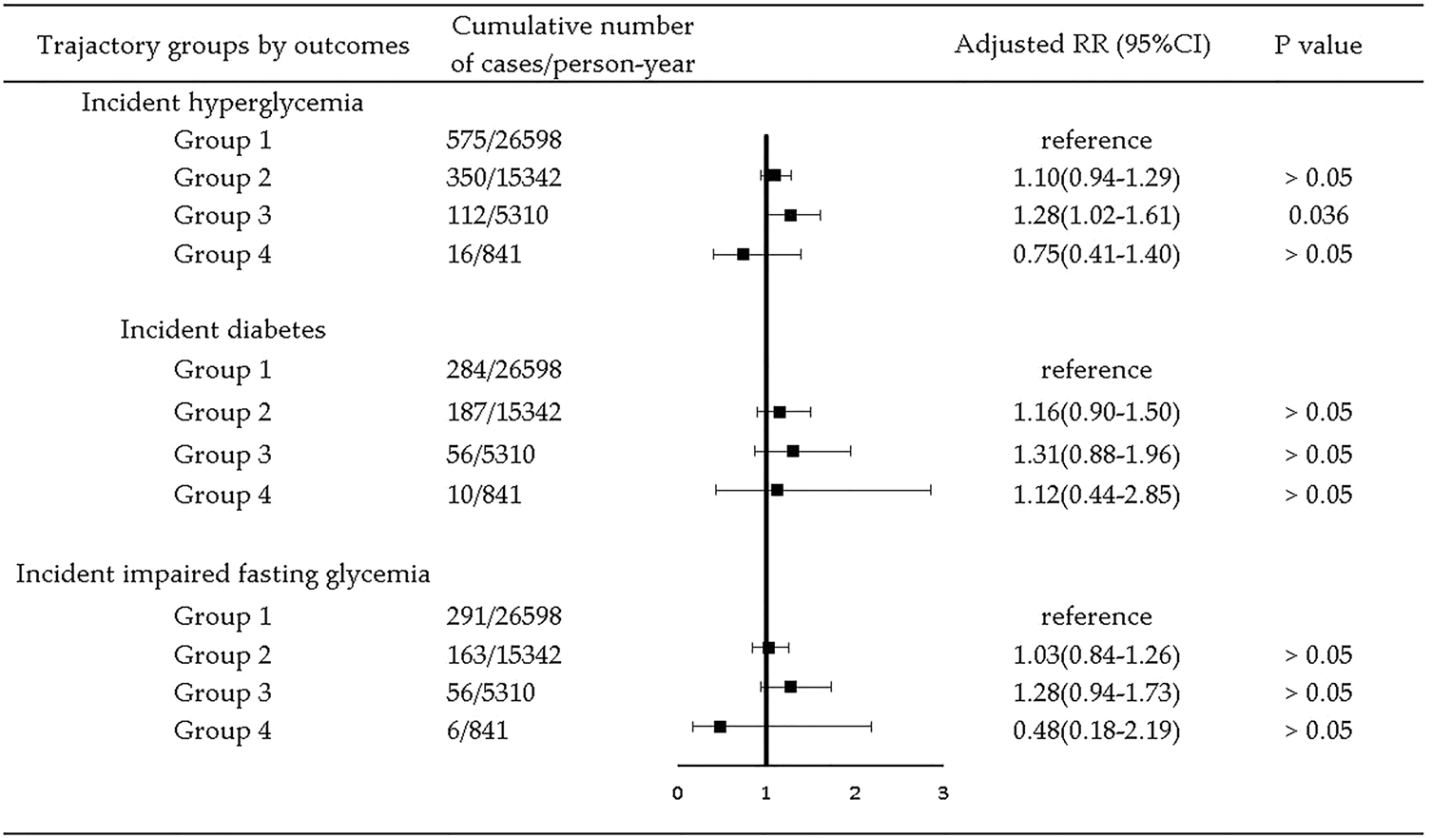
Association between trajectory groups and risk of hyperglycemia by type of outcome in all participants. Adjusted RR was yielded by adjustment for age, gender (categorical), marriage status(categorical), education level (categorical), geographic region (categorical), annual per capita household income, urbanicity index, physical activity (categorical), smoking (categorical), alcohol drinking (categorical), sleep duration (categorical), chronic disease history (categorical), total energy intake, CDGI (2019)-A score, BMI, WC, SBP, and DBP

**Fig. 3:**
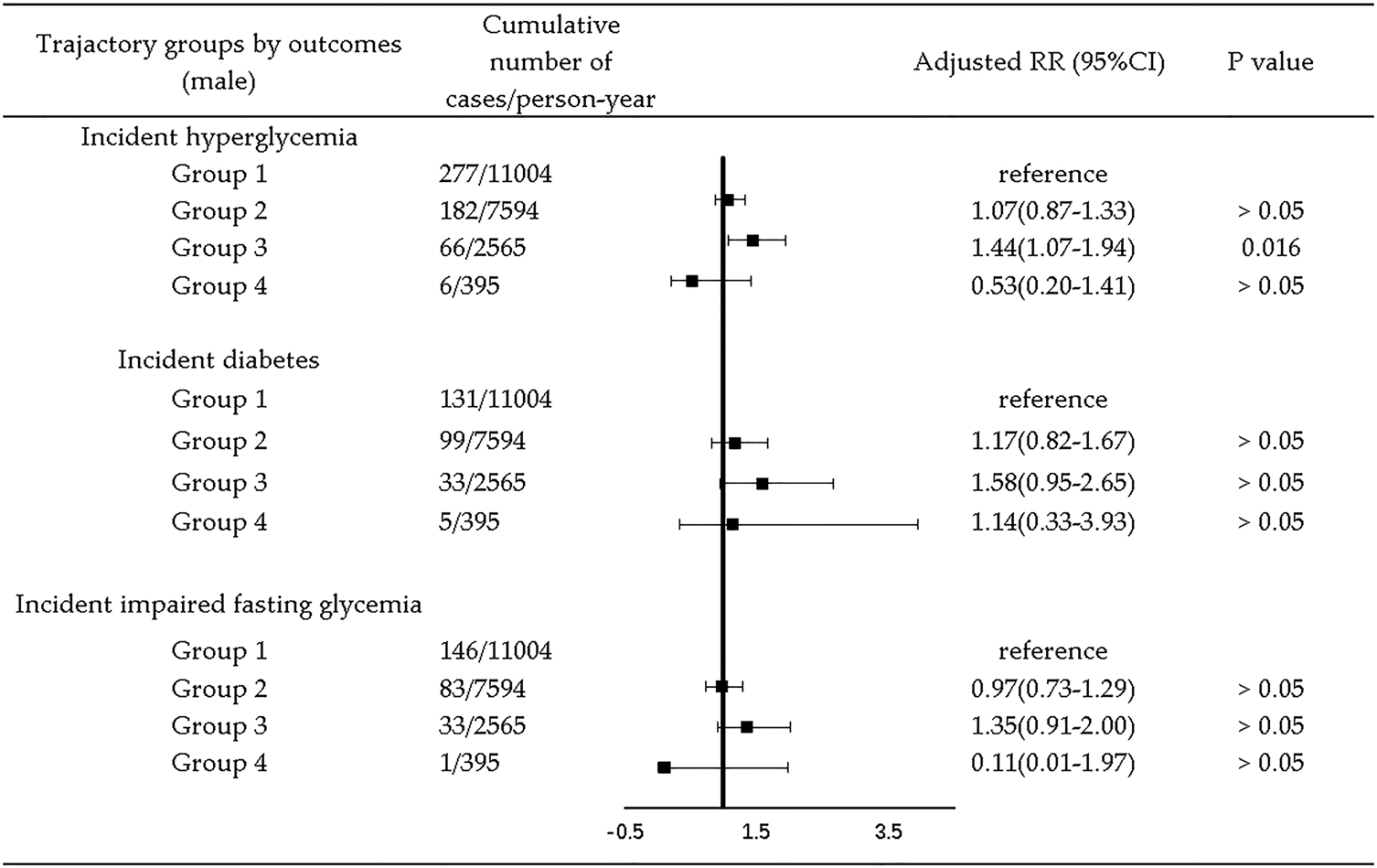
Association between trajectory groups and risk of hyperglycemia by type of outcome in males. Adjusted RR was yielded by adjustment for age, marriage status (categorical), education level (categorical), geographic region (categorical), annual per capita household income, urbanicity index, physical activity (categorical), smoking (categorical), alcohol drinking (categorical), sleep duration (categorical), chronic disease history (categorical), total energy intake, CDGI (2019)-A score, BMI, WC, SBP, and DBP

**Fig. 4:**
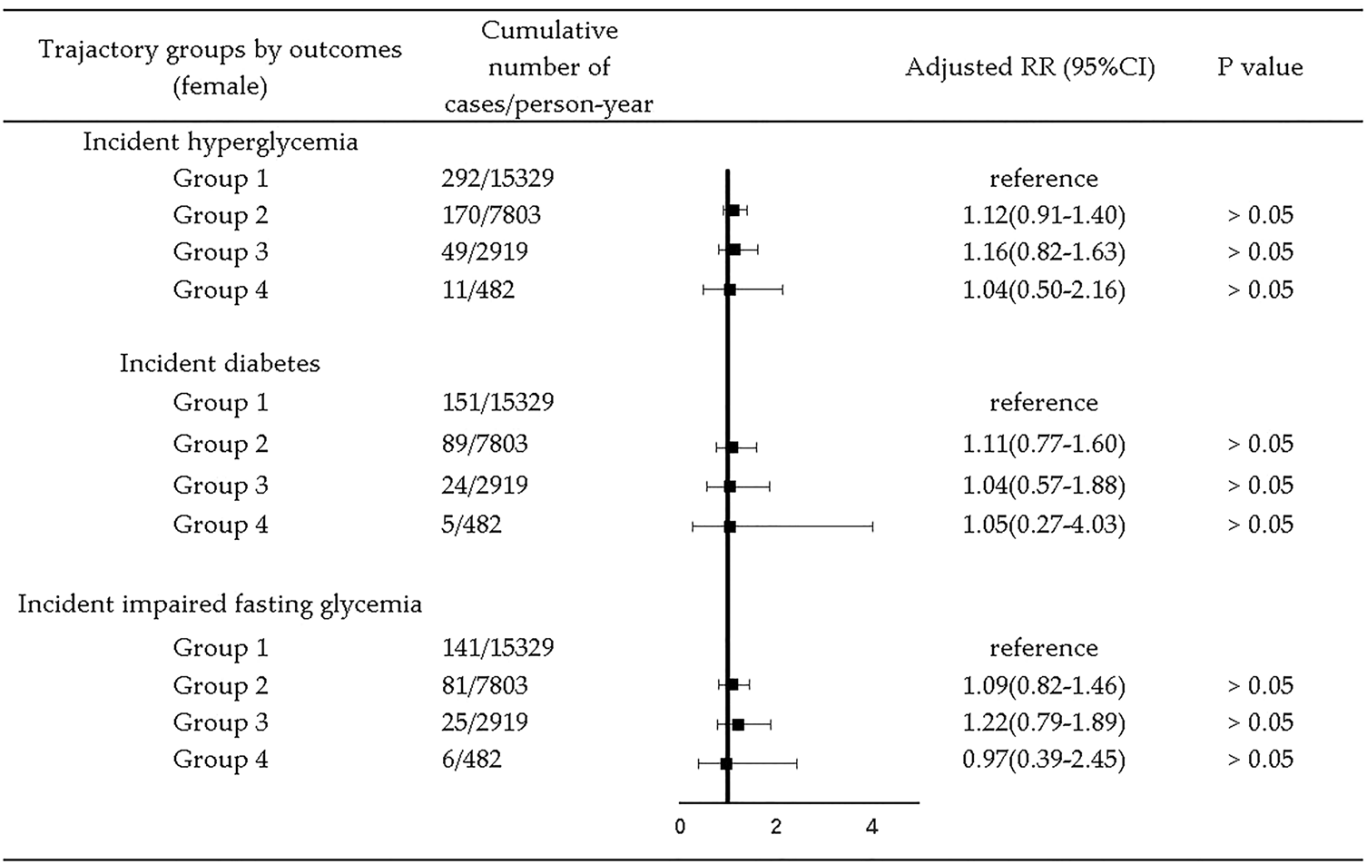
Association between trajectory groups and risk of hyperglycemia by type of outcome in females. Adjusted RR was yielded by adjustment for age, marriage status (categorical), education level (categorical), geographic region (categorical), annual per capita household income, urbanicity index, physical activity (categorical), smoking (categorical), alcohol drinking (categorical), sleep duration (categorical), chronic disease history (categorical), total energy intake, CDGI (2019)-A score, BMI, WC, SBP, and DBP

Among the 4711 participants, the median follow-up time was 7 years, ranging from 2 to 9 years. Numbers of outcome events for all trajectory groups in 9 years of follow-up are presented in Figs. 2, 3, 4.

In all the 9-year longitudinal analysis, Group 1 was taken as the reference group, because previous studies [11, 12, 23] suggested better cardiometabolic profiles associated with an energy balanced meal pattern. Among all participants, compared with Group 1, Group 3 were associated with higher risk of incident hyperglycemia (RR = 1.28, 95% CI = 1.02, 1.61). No association was found for incident diabetes and IFG (Fig. 2). Table S4 in supplemental materials presents detailed results of RR (95% CI) for Model 1 to Model 4.

Adjusted 9-year longitudinal analysis by gender showed that, in males, Group 3 was related to higher risk of hyperglycemia (RR = 1.44, 95% CI = 1.07, 1.94) compared with Group 1 (Fig. 3). In females, no relationship was found (Fig. 4). Table S5 in supplemental materials present detailed results of RR (95% CI) for Model 1–Model 4 for male and female.

In the sensitivity analyses, the identified trajectories were largely similar to those in the main analyses, and the subsequent risks of hyperglycemia by trajectory groups also yielded similar results as the main analyses. Figure S3 and Table S6 in supplemental materials present detailed results of sensitivity analysis.

## Discussion

In our study of Chinese adult population, we identified four distinct trajectory groups of energy intake distribution, characterized by “Energy evenly distributed with steady trend group” (Group 1); “Dinner and lunch energy dominant with relatively steady trend group” (Group 2); “Dinner energy dominant with increasing trend and breakfast energy with declining trend group” (Group 3); and “breakfast and dinner energy dominant with increasing trend group” (Group 4). Compared with Group 1, Group 3 was associated with higher risk of hyperglycemia in 9-year follow-up. Among males, Group 3 was associated with higher risks of hyperglycemia and diabetes in 9-year follow-up.

Few studies have assessed meal patterns based on energy intake distribution. Eicher-Miller et al. [12] and Aqeel et al. [23] identified temporal eating patterns based on energy contribution or absolute energy intake during an hourly time interval by using kernel k-means cluster analysis in the U.S. adult population. Using latent class analysis, Leech et al. [11] examined temporal eating patterns of Australian adults based on whether or not an eating occasion occurred within each hour of the day. We also examined the time-of-day of energy intake pattern of Chinese adults in a cross-sectional setting by latent class analysis in a previous study [24]. However, none of these studies investigated secular trend of energy intake contribution from meals. Two previous studies [25, 26] examined secular trend of distribution of daily intake of meals in the US and UK, respectively. Nevertheless, these studies were not able to capture heterogeneity in energy intake distribution, which can be represented by latent class or clustering method.

Using group-based multi-trajectory model, we found more than half of the participants (about 55.5%) were classified into one group following a steady 3:4:3 energy intake distribution pattern (Group 1). The sociodemographic and anthropometric profiles in this group were similar to those of the “energy balanced pattern” identified in Aqeel’s study [23] and the “conventional pattern” identified in Leech’s study [11]. One-third (about 32.3%) of participants in our study followed a generally steady 2:4:4 energy intake distribution pattern over years. Only about 12.2% of participants showed a relatively changing energy intake distribution pattern over years in our study. Specifically, about 10.4% of participants reduced their breakfast EI% and lunch EI%, while increasing dinner EI% over time (Group 3). Another small portion of 1.8% of participants had a sharp decline of EI% from lunch and an increase of EI% from breakfast and dinner over time (Group 4).

Different trajectory groups of energy intake distribution over a decade predicted the differential risks of incident hyperglycemia in our study. We found that participants Group 3 had significantly higher incidents of hyperglycemia, compared with Group 1 in 9 years of follow-up. Among the few prospective studies conducting on the topic of energy distribution and health outcomes, Bo et al. [10] examined the relationship of daily caloric intake at dinner and risk of hyperglycemia in a 6-year population-based prospective cohort, however, no significant relationship was found. Ren et al. [27] recently conducted a prospective analysis to examine the association between energy intake at dinner versus breakfast and risk of incidence of type 2 diabetes by cox regression using the CHNS (1997–2011). Results showed higher risk of incident type 2 diabetes in the 2nd, 3rd, 4th, and 5th quintile of difference in energy intake between dinner and breakfast, compared with the 1st quintile (HR = 1.38, 95%CI 1.10–1.72 for the 2nd quintile; HR = 1.46, 95%CI 1.13–1.87 for the 5th quintile). In contrast, our study did not find significant association between trajectory group and risk of incident diabetes in the whole participants in the long-term follow-up. Differences of results between our study and Ren’s study might be attributed to different study design, follow-up period, analysis methods applied, and covariates added in models.

Contrary to our anticipation, Group 2 characterized by dominant and steady EI% from dinner was not associated with higher risk of incident hyperglycemia in our study. A possible explanation might be that, although higher energy intake from dinner and lower energy intake from breakfast in Group 2 may lead to impaired glucose control compared with Group 1, but the effect may not be large enough in the long run to see significantly higher risk of hyperglycemia. By comparison, participants in Group 3 consumed over 40% of total energy at dinner, and the energy contribution of dinner showed an increasing trend over years in this group, which might play more significantly long-term effect on hyperglycemia risk, compared with Group 1. However, whether there is a threshold of dinner energy proportion that causes adverse effect on glucose control still need to be explored by more research.

We did not find any significant relationship in Group 4 consuming 40–50% energy from both dinner and breakfast. Nevertheless, because of the small sample size and small number of incident cases in this group, more research are needed to interpret the effect of this group.

When we identified trajectory groups by sex, similar trajectory groups were identified in males and females, indicating there was no distinct difference in energy intake distribution between sexes. However, when risks of outcomes were examined, Group 3 was found to be associated with higher risk of hyperglycemia in male, while no association was found in females. This result suggested energy intake distribution might have a deeper effect on male than on female in respect of developing hyperglycemia. However, more supporting evidence are needed before this conclusion can be made because rare evidence on sex disparity in this regard is available from other studies. Besides, ascertainment of outcomes in our study might also underestimate the risk of hyperglycemia in female. In our study, only fasting glucose was measured and used to define diabetes/IFG/hyperglycemia, no IGT was performed. However, other study has suggested that impairment in glucose disposal contributed to higher postprandial glucose concentrations in females than in males [28], as shown by epidemiological data [29–31] that a higher prevalence of IGT was found in females, while a higher prevalence of IFG was found in males. Therefore, lacking IGT might underestimate the incident rate of hyperglycemia in females in our study. Therefore, further studies with more laboratory biochemical indicators are needed to elucidate the sex disparity.

Compared with the previous studies [9, 10, 27, 32, 33] investigating the association between energy intake of one or two eating occasions assessed only once and the risk of diabetes, our study supported the result that higher energy intake in the evening and/or lower energy intake in the morning may be a contributor to dysglycemia. Moreover, our study added to this literature that compared with a steady and evenly distributed energy intake pattern, the energy distribution pattern with over 40% of energy intake from dinner with a rising trend over years, was associated with higher long-term risk of hyperglycemia. Our strategy to distinguish people at risk of hyperglycemia from the energy distribution pattern perspective might provide more effective recommendations for dietary guideline for diabetes prevention.

There are several potential underlying biological and physiological mechanisms behind the association between energy intake distribution and hyperglycemia. Under the regulation by the central clock system, postprandial glucose tolerance decreases in the biological evening in humans. Eating a significant amount of calorie later in the day leads to circadian misalignment between the central and peripheral clocks, which exerts a negative impact on glucose control [34]. Several mechanisms are suggested by studies: (1) pancreatic β-cell function reduces in the biological evening, leading to lower glucose tolerance in the biological evening [35]; (2) insulin resistance in peripheral tissues (liver, muscle, and adipose tissue) during circadian misalignment contributes to elevated postprandial glucose levels and reduced glucose tolerance [36]; (3) improper meal pattern might alter release of feeding-related gut hormones, such as glucagon-like peptide1 (GLP-1), which will result in circadian misalignment [37].

One of the strengths of our study was to identify distinct trajectory groups of energy intake distribution in a prospective, population-based setting, which adds to previous findings on meal pattern by using high-resolution data. Other strengths included long subsequent follow-up period, and inclusion of many related covariates. However, our study also had several limitations. Firstly, for the sake of model parsimony and convergence of multi-trajectory model, we did not consider random effects in the model, and assumed error variance was the same for all latent classes and all repeated time points [21], which was usually not the case in real situation. However, because our main interest was to obtain class-specific mean trajectories rather than individual variance, we thought it would not affect the trajectory group classification. Secondly, although we excluded participants with self-reported diabetes from 1997 to 2009 when trajectory groups were assessed, participants with IFG or IGT could not be excluded due to lack of blood sample collection in these years. But it was unlikely that this would affect the group classification, because the prevalence of diabetes and pre-diabetes is relatively low during 1990s and early 2000s in China [38]. Besides, we were not able to identify IFG in round 2011 and IGT from all rounds for the reason of no blood sample collection or no 2-h postprandial blood glucose test was done, which would possibly lead to an underestimation of risks in all groups. Thirdly, dietary assessment based on 3–24-h recalls was subject to recall bias. We excluded participants with extreme total energy intake in order to reduce this bias. And, although we adjusted for as many covariates as possible, the possibility of other confounders unable to be included in our study could not be ruled out. Lastly, the inclusion rate of the entire sample was relatively low due to the strict inclusion criteria for the study design, and the characteristics between participants included and excluded differed. This limited the generalizability of the study findings to the entire study participants.

## Conclusions

Four trajectory groups of energy intake distribution were identified in the Chinese population from 1997 to 2009. The “Dinner energy dominant with increasing trend and breakfast energy with declining trend group” was associated with higher risk of incident hyperglycemia in 9-year follow-up compared with “Energy evenly distributed with steady trend group”. Future studies are warranted to unravel the biological pathways of these associations, and to demonstrate the effectiveness of adjusting energy intake distribution to prevent hyperglycemia.

## Supplementary Information

Below is the link to the electronic supplementary material.

Supplementary file1 (DOCX 36 kb)

Supplementary file2 (PDF 678 kb)

## Data Availability

The datasets generated during and/or analyzed during the current study are available from the corresponding authors (B.Z.) on reasonable request.
