# Supplementary material for: Trajectories of energy intake distribution and subsequent risk of hyperglycemia among Chinese adults: findings from the China Health and Nutrition Survey (1997–2018)
Source: Eur J Nutr. 2021 Nov 27;61(3):1417–27. doi: 10.1007/s00394-021-02745-3 (PMC8921126; doi:10.1007/s00394-021-02745-3)
Supplement: Supplementary file 2 — Supplementary file2 (PDF 678 kb) [file 394_2021_2745_MOESM2_ESM.pdf]

Supplemental file 1

Submitted to **European Journal of Nutrition**

Trajectories of energy intake distribution and subsequent risk of hyperglycemia among Chinese adults：Findings from the China Health and Nutrition Survey (1997-2018)

Xiaoyun Song, Huijun Wang, Chang Su, Zhihong Wang, Wenwen Du, Haojie Hu, Feifei Huang, Jiguo Zhang, Xiaofang Jia, Hongru Jiang, Yifei Ouyang, Li Li, Jing Bai, Xiaofan Zhang, Gangqiang Ding, and Bing Zhang *

National Institute for Nutrition and Health, Chinese Center for Disease Control and Prevention, Beijing 100050, China; sxydljk@126.com (X.S.); wanghj@ninh.chinacdc.cn (H.W.); suchang@ninh.chinacdc.cn (C.S.); wangzh@ninh.chinacdc.cn (Z.W.); Duww@ninh.chinacdc.cn (W.D.); 15732683256@163.com (H.H.); huangff@ninh.chinacdc.cn (F.H.); zhangjg@ninh.chinacdc.cn (J.Z.); jiaxf@ninh.chinacdc.cn (X.J.); jianghr@ninh.chinacdc.cn (H.J.); ouyyf@ninh.chinacdc.cn (Y.O.); lili@ninh.chinacdc.cn (L.L.); Baijing@ninh.chinacdc.cn (J.B.); Zhangxf@ninh.chinacdc.cn (X.Z.); dinggq@chinacdc.cn (G.D.)

* Correspondence: zhangbing@chinacdc.cn, Tel.: +86-10-6623-7008, Fax: +86-10-6771-1813

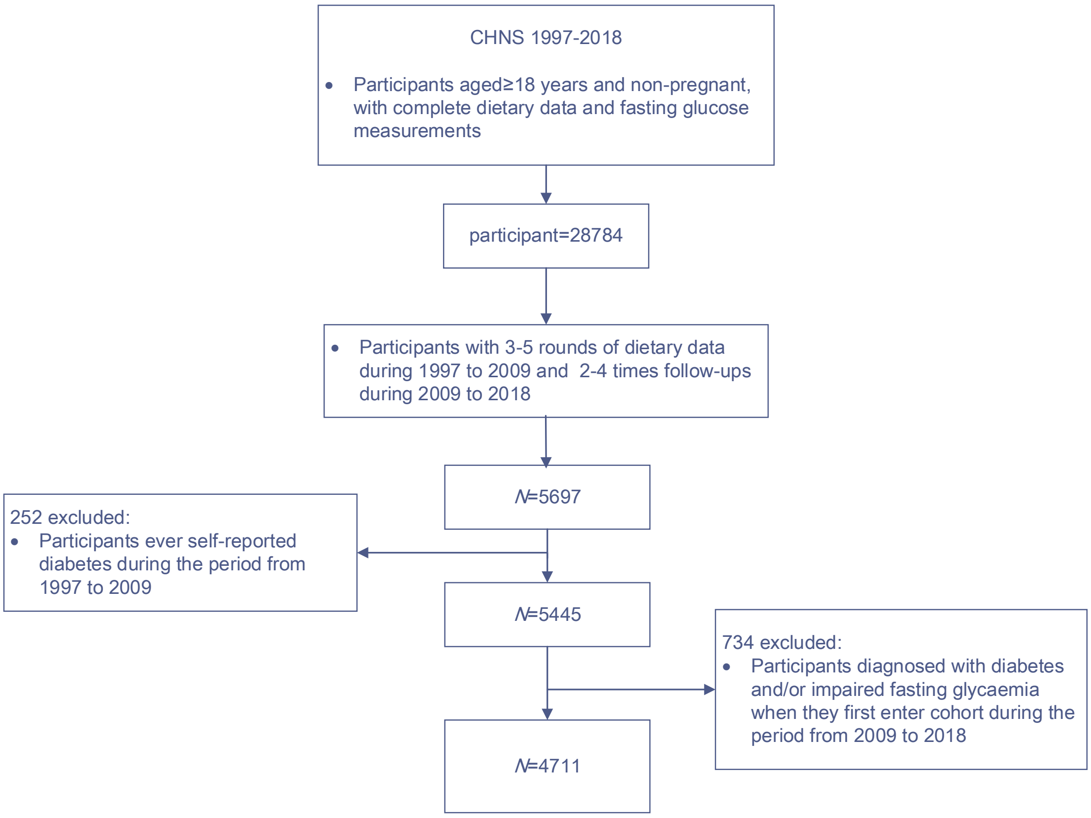

Figure S1. Flow chart of study population selection

Supplemental file 2

Submitted to **European Journal of Nutrition**

Trajectories of energy intake distribution and subsequent risk of hyperglycemia among Chinese adults：Findings from the China Health and Nutrition Survey (1997-2018)

Xiaoyun Song, Huijun Wang, Chang Su, Zhihong Wang, Wenwen Du, Haojie Hu, Feifei Huang, Jiguo Zhang, Xiaofang Jia, Hongru Jiang, Yifei Ouyang, Li Li, Jing Bai, Xiaofan Zhang, Gangqiang Ding, and Bing Zhang *

National Institute for Nutrition and Health, Chinese Center for Disease Control and Prevention, Beijing 100050, China; sxydljk@126.com (X.S.); wanghj@ninh.chinacdc.cn (H.W.); suchang@ninh.chinacdc.cn (C.S.); wangzh@ninh.chinacdc.cn (Z.W.); Duww@ninh.chinacdc.cn (W.D.); 15732683256@163.com (H.H.); huangff@ninh.chinacdc.cn (F.H.); zhangjg@ninh.chinacdc.cn (J.Z.); jiaxf@ninh.chinacdc.cn (X.J.); jianghr@ninh.chinacdc.cn (H.J.); ouyyf@ninh.chinacdc.cn (Y.O.); lili@ninh.chinacdc.cn (L.L.); Baijing@ninh.chinacdc.cn (J.B.); Zhangxf@ninh.chinacdc.cn (X.Z.); dinggq@chinacdc.cn (G.D.)

* Correspondence: zhangbing@chinacdc.cn, Tel.: +86-10-6623-7008, Fax: +86-10-6771-1813

Table S1. Comparison of characteristics between included and excluded participants, China Health and Nutrition Survey 1997-2018

Baseline characteristics	Excluded(n=24073)	Included(n=4711)	P value
Age (year, median [IQR])	43.6(30.6,57.7）	42.0(33.5,50.8)	<0.001
Gender (%)			0.163
Man	47.6	46.4	
Woman	52.4	53.6	
Marriage status (%)			<0.001
In marriage	76.0	88.5	
Other status	24.0	11.6	
Geographic region (%)			<0.001
Urban	43.2	27.7	
Rural	56.8	72.3	
Smoking (%)			<0.001
Nonsmoker	74.3	69.7	
Current smoker	25.7	30.3	
Drinking (%)			<0.001
nondrinker	65.9	63.0	
Current drinker	34.1	37.0	
Education level (%)			<0.001
Primary school	30.0	52.8	
Middle school	33.4	30.6	
High school and above	36.6	16.7	
Physical activity (%)			0.043
Low	95.2	95.6	
Medium	3.5	3.5	
High	1.3	0.9	
Chronic disease history (%)			<0.001
No	88.5	96.9	
Yes	11.5	3.1	
Per capita household income (yuan/year, median [IQR])	21329.6(8784.0,53462.6)	8900.8(4997.3,14309.9)	<0.001
Urbanicity score (median [IQR])	71.3 (51.4,85.7)	51.0 (36.8,69.2)	<0.001
BMI (mg/kg^2, mean [SD])	23.48(3.8)	22.3 (2.9)	<0.001
WC (cm, mean [SD])	81.4(11.2)	77.3(8.9)	0.134
SBP (mmHg, mean [SD])	122.1(18.2)	117.1(15.8)	<0.001
DBP (mmHg, mean [SD])	78.6(11.0)	76.3(10.4)	0.453

Supplemental file 3

Submitted to **European Journal of Nutrition**

Trajectories of energy intake distribution and subsequent risk of hyperglycemia among Chinese adults：Findings from the China Health and Nutrition Survey (1997-2018)

Xiaoyun Song, Huijun Wang, Chang Su, Zhihong Wang, Wenwen Du, Haojie Hu, Feifei Huang, Jiguo Zhang, Xiaofang Jia, Hongru Jiang, Yifei Ouyang, Li Li, Jing Bai, Xiaofan Zhang, Gangqiang Ding, and Bing Zhang *

National Institute for Nutrition and Health, Chinese Center for Disease Control and Prevention, Beijing 100050, China; sxydljk@126.com (X.S.); wanghj@ninh.chinacdc.cn (H.W.); suchang@ninh.chinacdc.cn (C.S.); wangzh@ninh.chinacdc.cn (Z.W.); Duww@ninh.chinacdc.cn (W.D.); 15732683256@163.com (H.H.); huangff@ninh.chinacdc.cn (F.H.); zhangjg@ninh.chinacdc.cn (J.Z.); jiaxf@ninh.chinacdc.cn (X.J.); jianghr@ninh.chinacdc.cn (H.J.); ouyyf@ninh.chinacdc.cn (Y.O.); lili@ninh.chinacdc.cn (L.L.); Baijing@ninh.chinacdc.cn (J.B.); Zhangxf@ninh.chinacdc.cn (X.Z.); dinggq@chinacdc.cn (G.D.)

* Correspondence: zhangbing@chinacdc.cn, Tel.: +86-10-6623-7008, Fax: +86-10-6771-1813

Table S2. Parameters of model-adequacy criteria of the multi-trajectory model

Trajectory group number	2ΔBIC	APPA	OCC	Entropy	Proportion of individuals in groups (%)
All participants (n=4711)					
1	NA	1	NA	NA	100.0
2	5074.6	0.92	9.9/14.1	0.738	58.8/41.2
3	4831.8	0.93	11.2/15.3/1692.5	0.846	57.9/39.7/2.4
**4**	**2289.6**	**0.92**	**11.3/17.52/73.5/2008.4**	**0.851**	**54.6/32.6/11.0/1.8**
5	1070.6	0.90	12.0/12.2/79.8/274.3/1670.2	0.847	47.4/36.9/10.7/3.3/1.8
Males (n=2188)					
1	NA	1	NA	NA	100.0
2	2258.1	0.92	10.4/12.4	0.728	55.7/44.32
3	2087.7	0.93	11.1/14.4/1806.7	0.840	55.6/41.9/2.5
**4**	**1057.5**	**0.92**	**12.3/15.9/75.7/7423.8**	**0.849**	**51.2/35.5/11.6/1.6**
5	No convergence				
Females (n=2523)					
1	NA	1	NA	NA	100.0
2	2691.0	0.93	9.9/15.2	0.747	61.1/38.9
3	2646.7	0.93	11.2/2828.1/16.5	0.852	59.8/2.3/37.9
**4**	**1137.8**	**0.92**	**73.4/10.6/18.9/6493.5**	**0.854**	**10.7/57.1/30.4/1.8**
5	585.0	0.90	2772.1/60.1/10.3/18.6/89.9	0.843	1.9/7.8/50.5/30.8/9.0

2ΔBIC, ≈the logged Bayes factor, > 10 was considered as a reasonable standard for strong evidence in favor of the complex model); APPA, average posterior probability of assignment, > 0.70 was indicative of a good model fit; OCC, odds of correct classification, > 5 for all groups was indicative of a good model fit; Entropy, >0.80 was indicative of better classification; Proportion of individuals in group, ≥ 1% for each group was accepted.

Supplemental file 4

Submitted to **European Journal of Nutrition**

Trajectories of energy intake distribution and subsequent risk of hyperglycemia among Chinese adults：Findings from the China Health and Nutrition Survey (1997-2018)

Xiaoyun Song, Huijun Wang, Chang Su, Zhihong Wang, Wenwen Du, Haojie Hu, Feifei Huang, Jiguo Zhang, Xiaofang Jia, Hongru Jiang, Yifei Ouyang, Li Li, Jing Bai, Xiaofan Zhang, Gangqiang Ding, and Bing Zhang *

National Institute for Nutrition and Health, Chinese Center for Disease Control and Prevention, Beijing 100050, China; sxydljk@126.com (X.S.); wanghj@ninh.chinacdc.cn (H.W.); suchang@ninh.chinacdc.cn (C.S.); wangzh@ninh.chinacdc.cn (Z.W.); Duww@ninh.chinacdc.cn (W.D.); 15732683256@163.com (H.H.); huangff@ninh.chinacdc.cn (F.H.); zhangjg@ninh.chinacdc.cn (J.Z.); jiaxf@ninh.chinacdc.cn (X.J.); jianghr@ninh.chinacdc.cn (H.J.); ouyyf@ninh.chinacdc.cn (Y.O.); lili@ninh.chinacdc.cn (L.L.); Baijing@ninh.chinacdc.cn (J.B.); Zhangxf@ninh.chinacdc.cn (X.Z.); dinggq@chinacdc.cn (G.D.)
* Correspondence: zhangbing@chinacdc.cn, Tel.: +86-10-6623-7008, Fax: +86-10-6771-1813

(a) Male

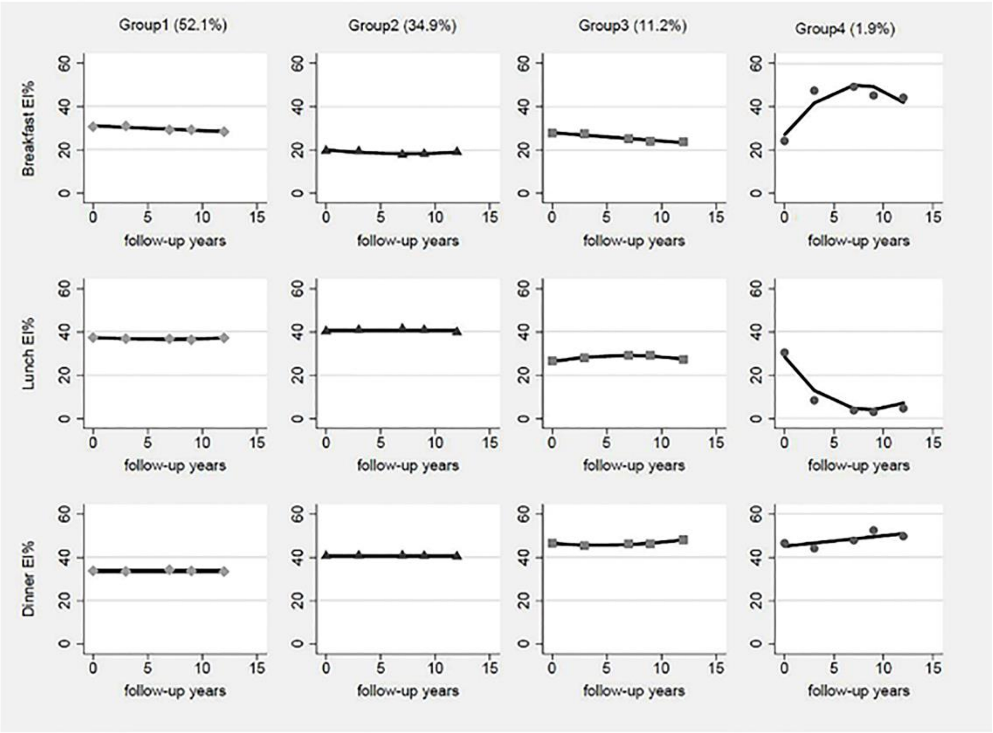

(b) Female

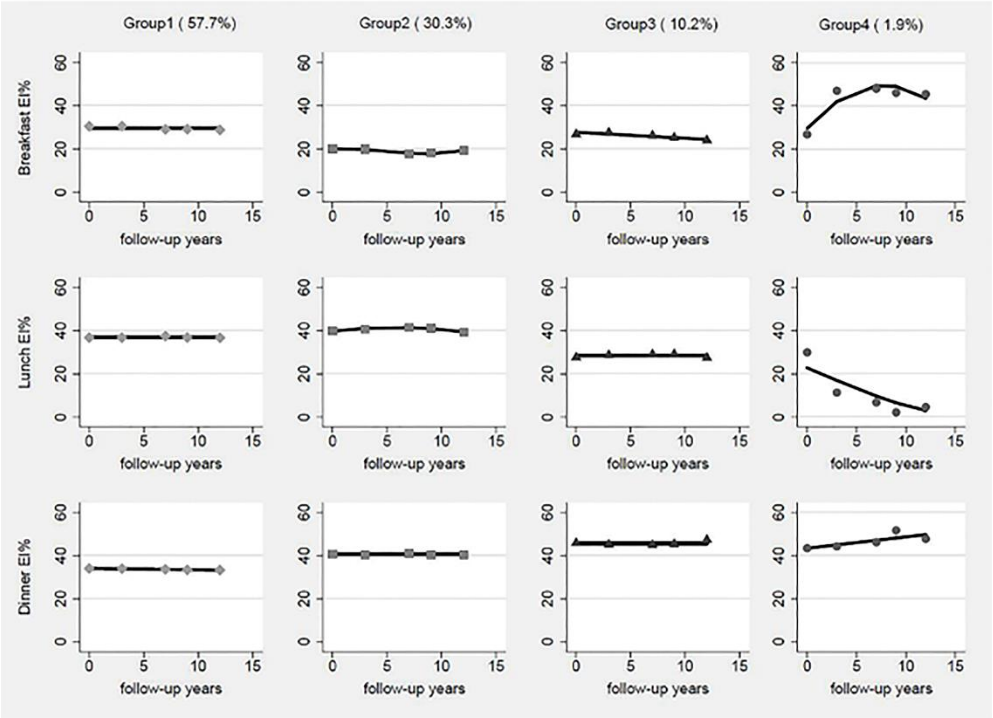

Figure S2. Estimated trajectory groups of energy intake distribution among (A)male participants and (B) female participants.

Group 1, labelled "Energy evenly distributed with steady trend group"; Group 2, labelled "Dinner and lunch energy dominant with relatively steady trend group"; Group 3, labelled "Dinner energy dominant with increasing trend and breakfast energy with declining trend group"; Group 4, labelled "breakfast and dinner energy dominant with increasing trend group".

Supplemental file 5

Submitted to **European Journal of Nutrition**

Trajectories of energy intake distribution and subsequent risk of hyperglycemia among Chinese adults：Findings from the China Health and Nutrition Survey (1997-2018)

Xiaoyun Song, Huijun Wang, Chang Su, Zhihong Wang, Wenwen Du, Haojie Hu, Feifei Huang, Jiguo Zhang, Xiaofang Jia, Hongru Jiang, Yifei Ouyang, Li Li, Jing Bai, Xiaofan Zhang, Gangqiang Ding, and Bing Zhang *

National Institute for Nutrition and Health, Chinese Center for Disease Control and Prevention, Beijing 100050, China; sxydljk@126.com (X.S.); wanghj@ninh.chinacdc.cn (H.W.); suchang@ninh.chinacdc.cn (C.S.); wangzh@ninh.chinacdc.cn (Z.W.); Duww@ninh.chinacdc.cn (W.D.); 15732683256@163.com (H.H.); huangff@ninh.chinacdc.cn (F.H.); zhangjg@ninh.chinacdc.cn (J.Z.); jiaxf@ninh.chinacdc.cn (X.J.); jianghr@ninh.chinacdc.cn (H.J.); ouyyf@ninh.chinacdc.cn (Y.O.); lili@ninh.chinacdc.cn (L.L.); Baijing@ninh.chinacdc.cn (J.B.); Zhangxf@ninh.chinacdc.cn (X.Z.); dinggq@chinacdc.cn (G.D.)

* Correspondence: zhangbing@chinacdc.cn, Tel.: +86-10-6623-7008, Fax: +86-10-6771-1813

Table S4. 9-year longitudinal analysis of effect of trajectory groups on the relative risk (RR) of hyperglycemia by type of outcome in all participants (n=4711) [1]

	Group 1 (n=2614)	Group 2 (n=1523)	Group 3 (n=490)	Group 4 (n=84)
	Risk ratio (95% CI)	Risk ratio (95% CI)	Risk ratio (95% CI)	Risk ratio (95% CI)
Incident hyperglycemia				
Model 1	1	1.05(0.90-1.21)	0.99(0.79-1.25)	0.85(0.51-1.42)
Model 2	1	1.00(0.85-1.17)	1.08(0.86-1.36)	0.84(0.48-1.48)
Model 3	1	0.99(0.84-1.15)	1.08(0.86-1.37)	0.83(0.47-1.46)
Model 4	1	1.10(0.94-1.29)	1.28(1.02-1.61) *	0.75(0.41-1.40)
Incident diabetes				
Model 1	1	1.13(0.89-1.42)	1.01(0.69-1.49)	1.06(0.49-2.27)
Model 2	1	1.03(0.80-1.33)	1.09(0.72-1.63)	1.13(0.47-2.72)
Model 3	1	1.02(0.79-1.33)	1.08(0.72-1.63)	1.12(0.46-2.70)
Model 4	1	1.16(0.90-1.50)	1.31(0.88-1.96)	1.12(0.44-2.85)
Incident impaired fasting glycemia				
Model 1	1	0.97(0.80-1.18)	0.99(0.74-1.33)	0.64(0.27-1.52)
Model 2	1	0.94(0.77-1.16)	1.11(0.82-1.49)	0.60(0.25-1.45)
Model 3	1	0.93(0.76-1.14)	1.11(0.83-1.50)	0.60(0.25-1.44)
Model 4	1	1.03(0.84-1.26)	1.28(0.94-1.73)	0.48(0.18-2.19)

[1] A three-level mixed-effects Poisson regression with robust (sandwich) estimation of variance, taking household as the third level, individual as the second level, and repeated measurements of individual as the first level. Model 1 adjusted for no covariates. Model 2 adjusted for age, gender (categorical), marriage status(categorical), an education level (categorical), geographic region (categorical), per capita household income, urbanicity index, physical activity (categorical), smoking (categorical), alcohol drinking (categorical), sleep duration (categorical), and chronic disease history (categorical). Model 3 additionally adjusted for total energy intake and CDGI (2019)-A score. Model 4 additionally adjusted for BMI, WC, SBP, and DBP. * $P < 0.05$, ** $P < 0.01$, **$P < 0.001$.

Supplemental file 6

Submitted to **European Journal of Nutrition**

Trajectories of energy intake distribution and subsequent risk of hyperglycemia among Chinese adults：Findings from the China Health and Nutrition Survey (1997-2018)

Xiaoyun Song, Huijun Wang, Chang Su, Zhihong Wang, Wenwen Du, Haojie Hu, Feifei Huang, Jiguo Zhang, Xiaofang Jia, Hongru Jiang, Yifei Ouyang, Li Li, Jing Bai, Xiaofan Zhang, Gangqiang Ding, and Bing Zhang *

National Institute for Nutrition and Health, Chinese Center for Disease Control and Prevention, Beijing 100050, China; sxydljk@126.com (X.S.); wanghj@ninh.chinacdc.cn (H.W.); suchang@ninh.chinacdc.cn (C.S.); wangzh@ninh.chinacdc.cn (Z.W.); Duww@ninh.chinacdc.cn (W.D.); 15732683256@163.com (H.H.); huangff@ninh.chinacdc.cn (F.H.); zhangjg@ninh.chinacdc.cn (J.Z.); jiaxf@ninh.chinacdc.cn (X.J.); jianghr@ninh.chinacdc.cn (H.J.); ouyyf@ninh.chinacdc.cn (Y.O.); lili@ninh.chinacdc.cn (L.L.); Baijing@ninh.chinacdc.cn (J.B.); Zhangxf@ninh.chinacdc.cn (X.Z.); dinggq@chinacdc.cn (G.D.)

* Correspondence: zhangbing@chinacdc.cn, Tel.: +86-10-6623-7008, Fax: +86-10-6771-1813

Table S4. 9-year longitudinal analysis of effect of trajectory groups on the relative risk (RR) of hyperglycemia by type of outcome in males and females [1]

	Group 1	Group 2	Group 3	Group 4
	Risk ratio (95% CI)	Risk ratio (95% CI)	Risk ratio (95% CI)	Risk ratio (95% CI)
Males(n=2188)	n=1139	n=764	n=244	n=41
Incident hyperglycemia				
Model 1	1	0.97(0.80-1.18)	1.07(0.81-1.42)	0.60(0.26-1.39)
Model 2	1	0.92(0.75-1.14)	1.26(0.94-1.69)	0.61(0.25-1.46)
Model 3	1	0.92(0.74-1.14)	1.27(0.95-1.70)	0.60(0.25-1.44)
Model 4	1	1.07(0.87-1.33)	1.44(1.07-1.94) *	0.53(0.20-1.41)
Incident diabetes				
Model 1	1	1.11(0.81-1.53)	1.15(0.72-1.81)	1.05(0.38-2.94)
Model 2	1	0.99(0.69-1.41)	1.38(0.82-2.31)	1.21(0.36-4.04)
Model 3	1	1.00(0.70-1.42)	1.37(0.82-2.30)	1.20(0.36-3.97)
Model 4	1	1.17(0.82-1.67)	1.58(0.95-2.65)	1.14(0.33-3.93)
Incident impaired fasting glycemia				
Model 1	1	0.84(0.64-1.10)	1.02(0.70-1.49)	0.19(0.03-1.31)
Model 2	1	0.85(0.64-1.13)	1.20(0.82-1.76)	0.19(0.03-1.21)
Model 3	1	0.84(0.63-1.11)	1.21(0.83-1.78)	0.19(0.03-1.21)
Model 4	1	0.97(0.73-1.29)	1.35(0.91-2.00)	0.11(0.01-1.97)
Females(n=2533)	n=1455	n=764	n=257	n=47
Incident hyperglycemia				
Model 1	1	1.12(0.91-1.38)	0.91(0.65-1.26)	1.15(0.59-2.23)
Model 2	1	1.07(0.86-1.33)	0.95(0.70-1.32)	1.14(0.57-2.30)
Model 3	1	1.05(0.85-1.31)	0.94(0.67-1.32)	1.12(0.55-2.26)
Model 4	1	1.12(0.91-1.40)	1.16(0.82-1.63)	1.04(0.50-2.16)
Incident diabetes				
Model 1	1	1.11(0.79-1.56)	0.82(0.47-1.43)	0.94(0.28-3.15)
Model 2	1	1.05(0.73-1.51)	0.82(0.45-1.49)	1.00(0.27-3.70)
Model 3	1	1.03(0.71-1.49)	0.81(0.45-1.48)	0.99(0.26-3.72)
Model 4	1	1.11(0.77-1.60)	1.04(0.57-1.88)	1.05(0.27-4.03)
Incident impaired fasting glycemia				
Model 1	1	1.11(0.84-1.47)	0.96(0.63-1.47)	1.31(0.55-3.15)
Model 2	1	1.04(0.78-1.39)	1.04(0.68-1.59)	1.23(0.51-2.98)
Model 3	1	1.03(0.78-1.38)	1.04(0.68-1.60)	1.20(0.49-2.90)
Model 4	1	1.09(0.82-1.46)	1.22(0.79-1.89)	0.97(0.39-2.45)

[1] A three-level mixed-effects Poisson regression with robust (sandwich) estimation of variance, taking household as the third level, individual as the second level, and repeated measurements of individual as

the first level. Model 1 adjusted for no covariates. Model 2 adjusted for age, marriage status(categorical), an education level (categorical), geographic region (categorical), per capita household income, urbanicity index, physical activity (categorical), smoking (categorical), alcohol drinking (categorical), sleep duration (categorical), and chronic disease history (categorical). Model 3 additionally adjusted for total energy intake and CDGI (2019)-A score. Model 4 additionally adjusted for BMI, WC, SBP, and DBP. * $P < 0.05$, ** $P < 0.01$, **$P < 0.001$.

Supplemental file 7

Submitted to **European Journal of Nutrition**

Trajectories of energy intake distribution and subsequent risk of hyperglycemia among Chinese adults：Findings from the China Health and Nutrition Survey (1997-2018)

Xiaoyun Song, Huijun Wang, Chang Su, Zhihong Wang, Wenwen Du, Haojie Hu, Feifei Huang, Jiguo Zhang, Xiaofang Jia, Hongru Jiang, Yifei Ouyang, Li Li, Jing Bai, Xiaofan Zhang, Gangqiang Ding, and Bing Zhang *

National Institute for Nutrition and Health, Chinese Center for Disease Control and Prevention, Beijing 100050, China; sxydljk@126.com (X.S.); wanghj@ninh.chinacdc.cn (H.W.); suchang@ninh.chinacdc.cn (C.S.); wangzh@ninh.chinacdc.cn (Z.W.); Duww@ninh.chinacdc.cn (W.D.); 15732683256@163.com (H.H.); huangff@ninh.chinacdc.cn (F.H.); zhangjg@ninh.chinacdc.cn (J.Z.); jiaxf@ninh.chinacdc.cn (X.J.); jianghr@ninh.chinacdc.cn (H.J.); ouyyf@ninh.chinacdc.cn (Y.O.); lili@ninh.chinacdc.cn (L.L.); Baijing@ninh.chinacdc.cn (J.B.); Zhangxf@ninh.chinacdc.cn (X.Z.); dinggq@chinacdc.cn (G.D.)

* Correspondence: zhangbing@chinacdc.cn, Tel.: +86-10-6623-7008, Fax: +86-10-6771-1813

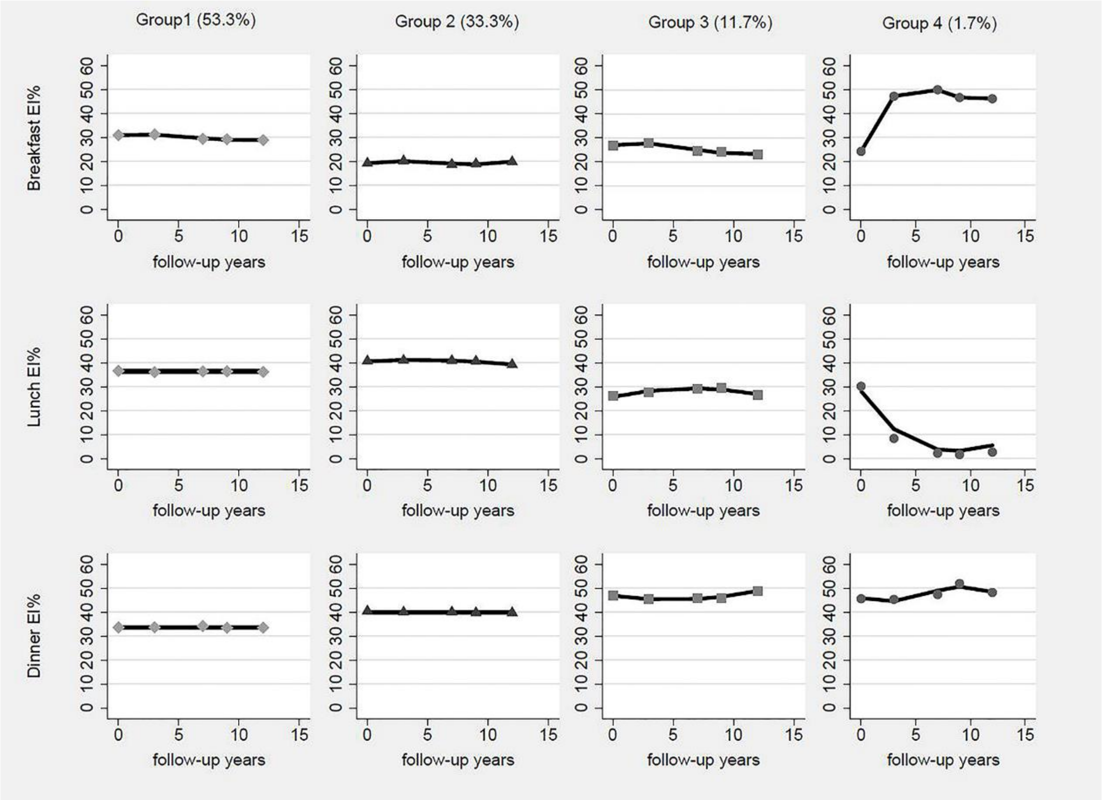

Figure S3. Estimated trajectory groups of energy intake distribution among Chinese adults with 3-5 rounds from 1997 to 2009 and all 4 rounds from 2009 to 2018 (n=1660)

Supplemental file 8

Submitted to **European Journal of Nutrition**

Trajectories of energy intake distribution and subsequent risk of hyperglycemia among Chinese adults：Findings from the China Health and Nutrition Survey (1997-2018)

Xiaoyun Song, Huijun Wang, Chang Su, Zhihong Wang, Wenwen Du, Haojie Hu, Feifei Huang, Jiguo Zhang, Xiaofang Jia, Hongru Jiang, Yifei Ouyang, Li Li, Jing Bai, Xiaofan Zhang, Gangqiang Ding, and Bing Zhang *

National Institute for Nutrition and Health, Chinese Center for Disease Control and Prevention, Beijing 100050, China; sxydljk@126.com (X.S.); wanghj@ninh.chinacdc.cn (H.W.); suchang@ninh.chinacdc.cn (C.S.); wangzh@ninh.chinacdc.cn (Z.W.); Duww@ninh.chinacdc.cn (W.D.); 15732683256@163.com (H.H.); huangff@ninh.chinacdc.cn (F.H.); zhangjg@ninh.chinacdc.cn (J.Z.); jiaxf@ninh.chinacdc.cn (X.J.); jianghr@ninh.chinacdc.cn (H.J.); ouyyf@ninh.chinacdc.cn (Y.O.); lili@ninh.chinacdc.cn (L.L.); Baijing@ninh.chinacdc.cn (J.B.); Zhangxf@ninh.chinacdc.cn (X.Z.); dinggq@chinacdc.cn (G.D.)

* Correspondence: zhangbing@chinacdc.cn, Tel.: +86-10-6623-7008, Fax: +86-10-6771-1813

Table S5. 9-year longitudinal analysis of effect of trajectory groups on the relative risk (RR) of hyperglycemia by type of outcome among participants with 3-5 rounds from 1997 to 2009 and all 4 rounds from 2009 to 2018 (n=1660) [1]

	Group 1 (n=884)	Group 2(n=553)	Group 3 (n=194)	Group 4 (n=29)
	Risk ratio (95% CI)	Risk ratio (95% CI)	Risk ratio (95% CI)	Risk ratio (95% CI)
Incident hyperglycemia				
Model 1	1	1.11(0.91-1.35)	1.19(0.91-2.56)	1.37(0.79-2.40)
Model 2	1	1.08(0.86-1.35)	1.29(0.96-1.72)	1.45(0.74-2.83)
Model 3	1	1.08(0.86-1.35)	1.30(0.97-1.74)	1.44(0.74-2.81)
Model 4	1	1.17(0.94-1.45)	1.51(1.12-2.02) **	1.27(0.65-2.46)
Incident diabetes				
Model 1	1	1.15(0.80-1.66)	1.25(0.76-2.07)	1.97(0.74-5.23)
Model 2	1	1.10(0.72-1.70)	1.42(0.78-2.56)	2.61(0.77-8.83)
Model 3	1	1.12(0.73-1.73)	1.43(0.79-2.58)	2.59(0.76-8.80)
Model 4	1	1.22(0.81-1.85)	1.66(0.93-2.96)	2.43(0.72-8.17)
Incident impaired fasting glycemia				
Model 1	1	1.05(0.82-1.36)	1.17(0.83-1.65)	0.98(0.38-2.57)
Model 2	1	1.02(0.78-1.34)	1.31(0.93-1.87)	0.93(0.36-2.42)
Model 3	1	1.01(0.77-1.32)	1.32(0.93-1.87)	0.94(0.36-2.43)
Model 4	1	1.09(0.84-1.42)	1.49(1.05-2.10) *	0.79(0.32-1.93)

[1] A three-level mixed-effects Poisson regression with robust (sandwich) estimation of variance, taking household as the third level, individual as the second level, and repeated measurements of individual as the first level. Model 1 adjusted for no covariates. Model 2 adjusted for age, gender (categorical), marriage status(categorical), an education level (categorical), geographic region (categorical), per capita household income, urbanicity index, physical activity (categorical), smoking (categorical), alcohol drinking (categorical), sleep duration (categorical), and chronic disease history (categorical). Model 3 additionally adjusted for total energy intake and CDGI (2019)-A score. Model 4 additionally adjusted for BMI, WC, SBP, and DBP. * $P < 0.05$, ** $P < 0.01$, **$P < 0.001$.
